# Photoprotective Acclimation of the *Arabidopsis thaliana* Leaf Proteome to Fluctuating Light

**DOI:** 10.3389/fgene.2020.00154

**Published:** 2020-03-05

**Authors:** Stefan Niedermaier, Trang Schneider, Marc-Oliver Bahl, Shizue Matsubara, Pitter F. Huesgen

**Affiliations:** ^1^ZEA-3 Analytics, Forschungszentrum Jülich, Jülich, Germany; ^2^IBG-2 Plant Sciences, Forschungszentrum Jülich, Jülich, Germany; ^3^iGRAD-Plant, Department of Biology, Heinrich Heine University Düsseldorf, Düsseldorf, Germany; ^4^Cologne Excellence Cluster on Cellular Stress Responses in Aging Associated Diseases (CECAD), Medical Faculty and University Hospital, University of Cologne, Cologne, Germany

**Keywords:** acclimation, fluctuating light, leaf proteome, photooxidative stress, photoprotection, protein turnover, time of day, quantitative proteomics

## Abstract

Plants are subjected to strong fluctuations in light intensity in their natural growth environment, caused both by unpredictable changes due to weather conditions and movement of clouds and upper canopy leaves and predictable changes during day-night cycle. The mechanisms of long-term acclimation to fluctuating light (FL) are still not well understood. Here, we used quantitative mass spectrometry to investigate long-term acclimation of low light-grown *Arabidopsis thaliana* to a FL condition that induces mild photooxidative stress. On the third day of exposure to FL, young and mature leaves were harvested in the morning and at the end of day for proteome analysis using a stable isotope labeling approach. We identified 2,313 proteins, out of which 559 proteins exhibited significant changes in abundance in at least one of the four experimental groups (morning-young, morning-mature, end-of-day-young, end-of-day-mature). A core set of 49 proteins showed significant responses to FL in three or four experimental groups, which included enhanced accumulation of proteins involved in photoprotection, cyclic electron flow around photosystem I, photorespiration, and glycolysis, while specific glutathione transferases and proteins involved in translation and chlorophyll biosynthesis were reduced in abundance. In addition, we observed pathway- and protein-specific changes predominantly at the end of day, whereas few changes were observed exclusively in the morning. Comparison of the proteome data with the matching transcript data revealed gene- and protein-specific responses, with several chloroplast-localized proteins decreasing in abundance despite increased gene expression under FL. Together, our data shows moderate but widespread alterations of protein abundance during acclimation to FL and suggests an important role of post-transcriptional regulation of protein abundance.

## Introduction

Earth’s terrestrial environment is highly dynamic and variable, with daily and seasonal changes in factors that affect plant life, such as light, temperature, and rainfall. Of all the environmental factors, light is the one that changes most rapidly and frequently due to Earth’s rotation, weather conditions, cloud movement, and foliage motion in the wind (swaying, fluttering). Oxygenic photosynthesis―the type of photosynthesis which uses water as the electron donor and produces O_2_ as a byproduct―must operate with this very erratic power supply which fluctuates between energy shortage and energy overload.

Plants have evolved manifold mechanisms to balance energy and electron supply and demand during light fluctuations. In a short term, for example, the supply-demand imbalance results in acidification of thylakoid lumen which triggers thermal dissipation of absorbed light energy (or non-photochemical quenching, NPQ) in light harvesting antenna complexes of photosystem II (PSII) *via* PsbS protein protonation (Li et al., 2004) and activation of violaxanthin de-epoxidase (Hager and Holocher, 1994). Low lumenal pH also downregulates electron transport by slowing down plastoquinol reoxidation of cytochrome b_6_f (Takizawa et al., 2007). On the acceptor side of photosystem I (PSI), excess electrons can be transferred from ferredoxin back to plastoquinone by cyclic electron flow (CEF) involving PGR5 (proton gradient regulation 5; Munekage et al., 2002) or NADH dehydrogenase (NDH)-like complex (Shikanai et al., 1998), thereby pumping H^+^ from chloroplast stroma into the lumen at cytochrome b_6_f and NDH-like complex without synthesizing NADPH. Chloroplasts also have various enzymatic and non-enzymatic antioxidants which detoxify reactive oxygen species (ROS) generated by inevitable transfer of excess light energy and excess electrons to O_2_ (Noctor and Foyer, 1998; Asada, 1999; Asensi-Fabado and Munne-Bosch, 2010). Even though these and other mechanisms are operating to protect the photosynthetic apparatus against excess light and photooxidation, PSII reaction center, the D1 protein in particular, is prone to photoinactivation and photodamage not only under excess light but also under low light (Anderson et al., 1997). Damaged PSII reaction centers are continuously repaired through intricate multi-step processes of disassembly, degradation of damaged D1 and insertion of newly synthesized D1 followed by reassembly to maintain the PSII activity and minimize chronic photoinhibition (Jarvi et al., 2015; Theis and Schroda, 2016). Since the repair of PSII is also sensitive to ROS, which inhibits D1 protein synthesis (Nishiyama et al., 2004), it is essential to keep ROS under control.

In a long term, acclimation to growth light environment alters a range of plant traits, from biochemical composition to morphology and architecture (Poorter et al., 2019), which together enhance plant performance and fitness. Some of the components of short-term mechanisms described above are known to be increased or decreased during long-term photoacclimation. Typically, accumulation of PSII light harvesting antenna complexes is increased under low light to compensate for limited light energy supply, whereas cytochrome b_6_f and ATP synthase are more abundant in high light to boost electron transport and ATP production (Anderson et al., 1988; Walters, 2005; Schottler and Toth, 2014). Leaves in high light also contain larger pools of antioxidants and have increased capacities for photoprotection and D1 repair (Aro et al., 1993; Grace and Logan, 1996; Demmig-Adams, 1998). Despite the wealth of knowledge about the phenotypic outputs of long-term photoacclimation, the molecular mechanisms, which control these variations, are elusive and we know little about how they are coordinated at different levels and time scales (Dietz, 2015).

Most laboratory experiments of long-term acclimation are conducted under non-fluctuating constant light (CL) conditions, even though these conditions differ greatly from highly variable natural light environments (Annunziata et al., 2017; Matsubara, 2018). So far, only a small number of studies have investigated the mechanisms of long-term acclimation to fluctuating light (FL) (Yin and Johnson, 2000; Alter et al., 2012; Annunziata et al., 2017; Vialet-Chabrand et al., 2017; Kaiser et al., 2018; Matsubara, 2018; Schneider et al., 2019). In order to gain a systems-level overview of long-term acclimatory changes happening in FL conditions, we recently studied global reprogramming of gene expression in young and mature leaves of low-light grown Arabidopsis plants after 3-d exposure to a highly dynamic FL regime (Schneider et al., 2019). Long-term acclimation to this kind of FL conditions, that are characterized by repetitive exposure to brief and strong light pulses (“lightflecks”) in otherwise light-limited environment, enhances NPQ and ROS scavenging capacities in Arabidopsis leaves while repressing PSII electron transport, sugar and starch accumulation, as well as growth (Alter et al., 2012; Schneider et al., 2019). The transcriptomic changes observed under the FL condition included, among others, upregulation of many genes involved in photosynthesis, photoprotection and photorespiration, or metabolism of antioxidants, pigments, prenylquinones, and lipids (Schneider et al., 2019). Importantly, the FL response of gene expression differed between young and mature leaves or in the same leaves between morning and the end of day, highlighting a strong influence of leaf development and time of day on long-term acclimation at the gene expression level. While the changes in gene expression were largely consistent with the phenotypic alterations observed under the FL condition (Schneider et al., 2019), long-term acclimation may also involve post-transcriptional mechanisms to adjust protein levels and composition. Identification of such post-transcriptional mechanisms may open up new possibilities to improve plant productivity in FL environments (Ort et al., 2015).

Thus, we performed a quantitative proteome analysis of young and mature leaves harvested in the morning and at the end of day under the same FL and CL conditions as in the transcriptome study by Schneider et al. (2019). Specifically, we asked the following questions: to what extent are changes in gene expression matched by corresponding changes in protein abundance during FL acclimation, and whether acclimatory adjustment of proteome is also dynamic, differing between young and mature leaves or between morning and at the end of day.

## Methods

### Plant Materials and Growth Conditions

The plant materials used in this study were harvested in the same experiments with identical growth conditions and light treatments as in the gene expression and phenotype study (Schneider et al., 2019). Briefly, seeds of *Arabidopsis thaliana* Columbia-0 (Col-0) were sown on moist soil (Pikier; Balster Einheitserdewerk; Fröndenberg, Germany) and incubated at 5°C in the dark. After 4 days, they were transferred to a climate chamber with a 12 h/12 h light/dark cycle and 23°C/18°C air temperature at 60% relative humidity. The intensity of photosynthetically active radiation provided by fluorescent tubes (Fluora L36 W/77; Osram, Munich, Germany) was approx. 75 µmol photons m^−2^ s^−1^ (constant light, CL). After two weeks, seedlings were transferred to larger pots (7 × 7 × 8 cm, one plant per pot) filled with soil (type ED 73; Balster Einheitserdewerk). After 2 to 3 more weeks of cultivation in the CL condition, plants were divided into two groups: a control group, which remained in the CL condition, and a treatment group, which was exposed to FL in which the light intensity was changing between ~75 µmol photons m^−2^ s^−1^ (the CL background) and ~1,000 µmol photons m^−2^ s^−1^ in the same climate chamber. The high light pulses (ca. 20 s duration) of the FL treatment were applied every 5 min by using white LEDs (IP65, as-Schwabe, Eutingen, Germany) which were moving over the plants during the light period, as described previously (Alter et al., 2012). The light spectra of fluorescent tubes (CL) and white LEDs (high light pulses of FL) measured in the climate chamber using SpectraPen mini (Photon Systems Instruments, Drásov, Czech Republic) are shown in **Supplementary Figure 1**. Young and mature leaves were separately harvested from five individual plants (n = 5) in the morning (1 h after the light was turned on) and at the end of day (1 h before the light was turned off) on the third day of the FL or CL treatment as described by Schneider et al. (2019). Leaves were shock-frozen in liquid nitrogen and stored at −80°C until protein extraction.

### Sample Preparation

Samples were prepared by using our leaf extraction protocol for protein N-termini profiling in complex samples (Demir et al., 2017) with some modifications for shotgun proteomics. Frozen leaf samples were transferred into 5 ml reaction tubes and homogenized with 1.5 ml protein extraction buffer (150 mM HEPES, 6 M GuHCl, and 5 mM EDTA, pH 7.5 adjusted with 1 M NaOH) and added HALT Protease Inhibitor Cocktail (1:100, Thermo Fisher Scientific) using a KINEAMTICA Polytron at approx. 30.000 rpm for 1 min. The resulting suspension was filtered through a Miracloth mesh, the flow-through was collected and centrifuged for 10 min at 500 × g and 4°C and 1 ml of the supernatant was transferred to fresh 2 ml reaction tubes. A sample of 200 µl was precipitated using chloroform/methanol precipitation (Wessel and Flügge, 1984), washed twice with 1 ml ice-cold MeOH and dried at room temperature for 30 min before re-suspending in 200 µl protein extraction buffer. Twenty-five µl of each sample were taken to estimate protein concentration by BCA Protein Assay (Thermo Fisher Scientific) and also to verify intactness of proteome by visualizing on silver-stained SDS-PAGE (Laemmli, 1970; Blum et al., 1987). Cysteine residues were carbamidomethylated by adding 10 mM DTT for 1 h at 55°C, followed by 30 mM IAA for 30 min at 20°C in the dark and 15 mM DTT for 20 min at 37°C. Samples were precipitated again using chloroform/methanol, re-suspended in 100 mM HEPES pH 8.0 to a protein concentration of approx. 1 mg/ml based on the measurement results of the BCA assay and digested with trypsin at a protease:protein ratio of 1:100 at 37°C for 16 h. The efficiency of trypsin digestion was verified by silver-stained SDS-PAGE, and peptides were isotopically labeled by reductive dimethylation with 40 mM formaldehyde (standard ^12^CH_2_O formaldehyde for CL samples and heavy ^13^CD_2_O formaldehyde for FL samples) and 20 mM cyanoborohydride for 90 min at 20°C (Boersema et al., 2009). The reaction was quenched for 15 min by adding 100 mM TRIS pH 7.5. Subsequently, FL and CL samples were combined pairwise, resulting in five biological replicates of FL/CL comparison for every experimental group (morning-young, MY; morning-mature, MM; end of day-young, EY; end of day-mature, EM). Prior to LC-MS/MS, all samples were desalted using reversed-phase StageTips (Rappsilber et al., 2007).

### Mass Spectrometry, Protein Identification, and Quantification

Desalted peptides (500 ng), as estimated from the initial BSA assay, were loaded onto a C18 reverse phase capillary trap column (Acclaim PepMap C18, 50 cm column length, 75 µm × 2 cm, 3 µm particle size; Thermo Fisher Scientific) followed by separation on a C18 reverse phase analytical column (Acclaim PepMap C18, 75 µm × 50 cm, 2 µm particle size, 100 Å; Thermo Fisher Scientific) with an UltiMate3000 nano RSLC system (Thermo Fischer Scientific). Peptides were eluted with a 90-min gradient from 2% to 35% (v/v) acetonitrile in 0.1% formic acid in UPLC-grade H_2_O at a flow rate of 300 nl min^−1^. The nano-LC system was online coupled to an Impact II high-resolution quadrupole-time of flight (Q-TOF) tandem mass spectrometer (Bruker) using a CaptiveSpray nano-electrospray source (Bruker) as described (Beck et al., 2015; Weng et al., 2019). Peptides were identified, quantified, and matched to corresponding proteins using the Max-Quant software package, version 1.5.6.5. (Cox and Mann, 2008). Generic settings for Bruker Q-TOF instruments were used to match mass spectra to protein sequence in the *A. thaliana* Uniprot proteome database (www.uniprot.org; release 2016/11, 33463 entries). Trypsin was set as digestion protease allowing for up to two missed cleavages. The re-quantification feature of MaxQuant was enabled, “match between runs” was disabled. Lys side chains and peptide N-terminal dimethyl labeling was set according to the stable isotope reagents used (+28.0313 kDa for CL samples and +34.0631 kDa for FL samples). Carbamidomethylation of cysteines was set as fixed modification, N-terminal protein acetylation and methionine oxidation were considered as variable modifications with a maximum of five variable modifications per peptide.

### Data Analysis

The MaxQuant output file “proteingroups.txt” was loaded into Perseus software framework (Version 1.6.2.2) (Tyanova and Cox, 2018). Proteins annotated as potential contaminants, reverse hits (false positives) or only identified by modification site were removed, resulting in 2,313 identified protein groups. For each biological replicate of each experiment, protein abundance ratios (FL/CL) were log2 transformed and normalized to the median ratio of all proteins within the replicate to correct for potential differences in overall proteome abundance. Each of the four experimental groups (MY, MM, EY, EM) was filtered to select only those proteins that were quantified in at least three of the five replicates and the resulting data were imported into R statistical software to perform a moderated t-test using LIMMA package (Ritchie et al., 2015; Phipson et al., 2016). The results of each experimental group were reimported into Perseus, merged by Protein ID before adding Uniprot annotations (2018). Annotations of proteins and genes included in **Tables 1****–****3** and **Supplementary Tables 6**, **7**, and **9** were manually checked using TAIR10 gene description (https://www.arabidopsis.org/). Proteins with a Benjamini-Hochberg false discovery rate (FDR) corrected p-value ≤ 0.05 were considered to respond significantly to FL.

**Table 1:** Proteins with significant changes in protein abundance in FL compared to CL in all samples (MY, MM, EY, and EM). Significant changes are indicated in bold numbers.

Majority protein IDs	AGI locus	Gene names	Protein annotation	MY log_2_(FL/CL)	MM log_2_(FL/CL)	EY log_2_(FL/CL)	EM log_2_(FL/CL)	Peptides
O65581	AT4G26530	FBA5	fructose-bisphosphate aldolase 5, cytosolic	**0.62**	**0.48**	**0.62**	**0.41**	19
O81439	AT4G04020	FBN1A; PAP1	fibrillin family protein; plastid-lipid-associated protein 1	**0.55**	**0.29**	**0.67**	**0.39**	16
O82392	AT2G29630	THIC	hydroxymethylpyrimidine phosphate synthase; thiamine biosynthesis protein C	**0.36**	**0.29**	**0.26**	**0.21**	18
Q94B78; B3H5Y8	AT4G33010	GLDP1	glycine decarboxylase P protein 1	**0.32**	**0.30**	**0.35**	**0.19**	43
F4K410; F4K409	AT5G13650	SVR3	putative TypA-like translation elongation GTPase; suppressor of variegation 3	**0.29**	**0.42**	**0.32**	**0.24**	20
Q04836; Q04836-2	AT4G24770	RBP31	31 kDa RNA-binding protein, chloroplastic	**0.22**	**0.25**	**0.25**	**0.25**	8
Q8L7C9	AT1G78370	GSTU20	glutathione S-transferase U20	**−0.27**	**−0.42**	**−0.63**	**−0.51**	11
P42761	AT2G30870	GSTF10; ERD13	glutathione S-transferase F10; early response to dehydration 13	**−0.35**	**−0.36**	**−0.41**	**−0.37**	9

**Table 2:** Proteins with significant responses to FL in three of the four samples (MY, MM, EY, and EM). Significant changes are indicated by bold numbers.

Majority protein IDs	AGI locus	Gene names	Protein names and descriptions		MY log_2_(FL/CL)	MM log_2_(FL/CL)	EY log_2_(FL/CL)	EM log_2_(FL/CL)	Peptides
Q94AG1	AT3G07470			putative transmembrane protein (DUF538)	**1.06**	0.19	**1.06**	**0.88**	5
Q00218; F4JIZ3	AT4G33510	DHS2		3-deoxy-D-arabino-heptulosonate 7-phosphate synthase 2	**0.27**	0.38	**0.58**	**0.54**	14
Q94A68	AT1G06690			NAD(P)-linked oxidoreductase superfamily protein, chloroplastic	**0.33**	0.31	**0.40**	**0.40**	10
Q2V2S7	AT4G37925	NDHM		NDH-like complex subunit M	**0.34**	0.42	**0.37**	**0.33**	9
Q8L7S8; Q8L7S8-2; F4K180	AT5G26742	RH3; EMB1138		DEAD box ATP-dependent RNA helicase 3, chloroplastic; embryo defective 1138	0.23	**0.42**	**0.27**	**0.33**	26
O49629	AT4G22240	FBN1B; PAP2		fibrillin family protein; plastid-lipid-associated protein 2, chloroplastic	**0.37**	0.10	**0.59**	**0.32**	9
Q9FI56	AT5G50920	CLPC1		ATP-dependent Clp protease ATP-binding subunit C homologue 1	**0.24**	**0.28**	0.14	**0.28**	43
P56772	ATCG00670	clpP		ATP-dependent Clp protease plastid-encoded proteolytic subunit	0.10	**0.33**	**0.19**	**0.27**	4
F4KDZ4; Q9ZP05; A8MRP1; B3H560	AT5G09660	PMDH2		malate dehydrogenase 2, peroxisomal	0.03	**0.27**	**0.33**	**0.25**	17
Q56YA5	AT2G13360	SGAT		serine-glyoxylate aminotransferase	**0.27**	0.38	**0.36**	**0.24**	22
Q9LK47	AT3G23700	SRRP1		S1 RNA-binding ribosomal protein 1	**0.32**	0.28	**0.31**	**0.23**	11
Q9SJU4; F4IGL5; F4IGL7	AT2G21330	FBA1		fructose-bisphosphate aldolase 1, chloroplastic	**0.29**	0.21	**0.35**	**0.23**	19
P17745	AT4G20360	RABE1B; SVR11		translation elongation factor Tu, chloroplastic; suppressor of variagation 11	**0.24**	0.21	**0.21**	**0.22**	22
Q9SHG8	AT1G17100	HBP1		heme-binding protein 1	**0.36**	0.17	**0.32**	**0.22**	8
P25819; F4JM86	AT4G35090	CAT2		catalase 2	**0.29**	0.28	**0.31**	**0.21**	21
Q9MA79	AT1G43670	CFBP		fructose-1,6-bisphosphatase, cytosolic	**0.37**	0.22	**0.29**	**0.20**	15
P54150	AT4G25130	MSR4		peptide methionine sulfoxide reductase A4, chloroplastic	**0.25**	0.22	**0.27**	**0.19**	5
O65396	AT1G11860	GDCST		glycine decarboxylase T protein	**0.27**	0.09	**0.23**	**0.18**	22
Q9SA52	AT1G09340	CSP41B		stem-loop RNA binding protein of 41 kDa B, chloroplastic	**0.21**	0.12	**0.24**	**0.17**	22
O80448	AT2G38230	PDX1.1		pyridoxal 5’-phosphate synthase subunit 1.1	**0.37**	**0.34**	**0.31**	0.16	16
Q6NPR7	AT1G29470			S-adenosyl-L-methionine-dependent methyltransferases superfamily protein	**−0.37**		**−0.20**	**−0.18**	8
Q39255	AT1G75950	SKP1A		SKP1 homologue 1A	**−0.22**		**−0.23**	**−0.18**	3
O04487	AT1G09640			translation elongation factor EF1B, gamma chain 1	**−0.31**	−0.22	**−0.26**	**−0.20**	16
O04151;F4I529	AT1G56340	CRT1; CRT1A		calreticulin 1; calreticulin 1A	**−0.37**	−0.20	**−0.36**	**−0.22**	12
P56780	ATCG00710	psbH		10 kDa PSII reaction center phosphoprotein H	**−1.08**	−0.40	**−0.48**	**−0.25**	1
Q9LIA8	AT3G29360	UGD2		UDP-glucose 6-dehydrogenase 2	**−0.34**	−0.29	**−0.35**	**−0.26**	15
Q9SIN5	AT2G42530	COR15B		cold-regulated 15B, chloroplastic	**−0.62**	−0.30	**−0.26**	**−0.27**	10
O80852;O80852-2	AT2G30860	GSTF9		glutathione S-transferase F9	**−0.36**	−0.28	**−0.40**	**−0.28**	8
O82514;F4KAP2	AT5G63400	ADK1		adenylate kinase 1	**−0.81**	−0.24	**−0.31**	**−0.29**	10
Q96252	AT5G47030			ATP synthase F1 subunit delta’, mitochondrial	**−0.47**	**−0.40**		**−0.29**	3
P42794; P42794-2; P42795	AT2G42740; AT3G58700; AT4G18730; AT5G45775	RPL16A; RPL16B		60S ribosomal protein L16A; L16B	−0.10	**−0.32**	**−0.35**	**−0.30**	8
A8MSC5; O22263; F4IL52; F4IL53	AT2G47470	PDI11		protein disulfide isomerase 11	−0.21	**−0.39**	**−0.33**	**−0.33**	11
Q9LUT2	AT3G17390	MAT4		methionine adenosyltransferase 4	**−0.25**	−0.32	**−0.35**	**−0.38**	20
Q42547; B9DG18; F4HUL6; Q2V4M4	AT1G20620	CAT3		catalase 3	**−0.59**	−0.39	**−0.59**	**−0.38**	20
P21218	AT4G27440	PORB		NADPH:protochlorophyllide oxidoreductase B	**−0.33**	−0.24	**−0.28**	**−0.42**	20
Q9SXS7; F4K5T1	AT5G28020	CYSD2		cysteine synthase D2	**−0.38**	−0.35	**−0.48**	**−0.47**	5
F4HST2; P42759	AT1G20450	ERD10; LTI29		early response to dehydration 10; low temperature-induced 29	**−0.69**	−0.33	**−0.46**	**−0.52**	8
Q9ZPZ4	AT1G09310			protein of unknown function (DUF538)	**−0.48**	−0.46	**−0.53**	**−0.67**	10
Q945Q5	AT2G30695			bacterial trigger factor	0.10	**−0.84**	**−0.85**	**−0.71**	6
P31168	AT1G20440	COR47		dehydrin; cold-regulated 47	**−0.54**	−0.77	**−0.77**	**−0.72**	8
Q8RWG5	AT2G16630	FOCL1		fused outer cuticular ledge 1; pollen Ole e 1 allergen and extensin family protein	**−2.08**	**−2.21**	**−2.31**		1

**Table 3:** Proteins and genes with contrasting FL responses of protein and transcript abundance in mature leaves at the end of day. Localization annotation is the consensus listed in the SUBA database. Significant changes in abundance are indicated by bold letters.

Majority protein IDs	AGI locus	Gene names	Protein annotation	Localization	protein log2 (FL/CL)	transcript log2 (FL/CL)
P10796	AT5G38430	RBCS1B	Rubisco small subunit 1B	plastid	**−0.24**	**0.56**
P25856	AT3G26650	GAPA1	glyceraldehyde-3-phosphate dehydrogenase A subunit 1, chloroplastic	plastid	**−0.12**	**0.49**
Q9LW85	AT3G16000	MFP1	MAR-binding filament-like protein 1	plastid	**−0.20**	**0.44**
Q9SYL9	AT1G78630	EMB1473	50S ribosomal protein L13 family protein, chloroplastic	plastid	**−0.21**	**0.50**
F4IFC5	AT2G04842	EMB2761	threonyl-tRNA synthetase, chloroplastic/mitochondrial	plastid, mitochondria	**−0.13**	**0.51**
F4K329	AT5G42765		plasma membrane fusion protein	plastid	**−0.28**	**0.52**
Q945Q5	AT2G30695		bacterial trigger factor	plastid	**−0.71**	**0.64**
Q94BZ0	AT1G50450		saccharopine dehydrogenase	plastid	**−0.36**	**0.75**
A8MR78; Q8RUW5; Q8RUW5-2; Q3EBW0; F4IKK4; A8MQW0	AT2G22990	SNG1; SCPL8	sinapoylglucose:malate O-sinapoyltransferase	extracellular	**−0.42**	**1.40**

### GO Term Enrichment Analysis

Gene Ontology (GO) term and KEGG pathway enrichment analysis was performed using g:Profiler (Raudvere et al., 2019) with generic settings and whole proteome *A. thaliana* background list. ATG identifiers were used to run queries. If a protein referred to more than one gene, for example ribosomal proteins, only the first gene ID was considered. Significance thresholds were calculated using the multiple-testing corrected SCS algorithm and set to 0.05 FDR. If the number of enriched GO terms exceeded five, only the five most significant ones were shown.

## Results

**Figure 1:**
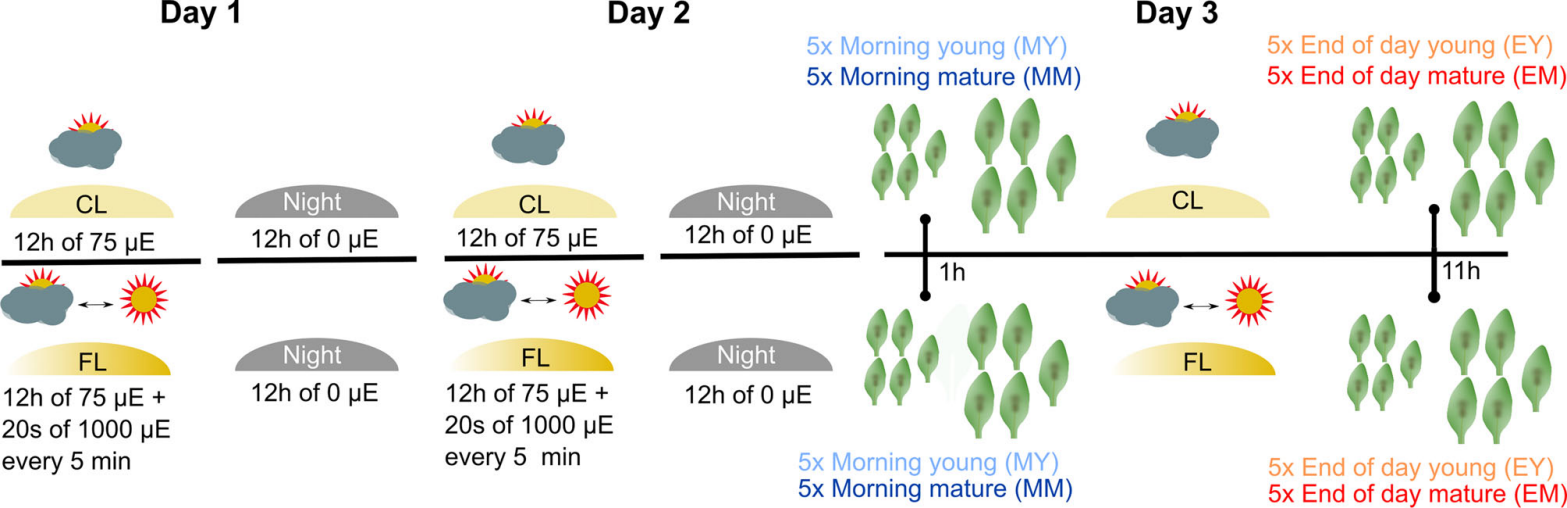
Experimental design and profiling AT proteome under FL. Six-week-old Arabidopsis plants grown at constant 75 µmol m^-2^ s^-1^ light intensity (CL) were split into a control group staying in CL and a group that was exposed to high intensity light pulses of 1,000 µmol m^-2^ s^-1^ for 20 seconds every 5 minutes under CL (fluctuating light, FL). On the third day of treatment, young and mature leaves were harvested 1 h after light on (morning young, MY; morning mature, MM) and 11 h after light on (end of day young, EY; end of day mature, EM), which we define as four “samples”. To obtain five biological replicates of each sample, proteomes were extracted from leaves of five independent plants.

To investigate the impact of FL on Arabidopsis leaf proteome, 4- to 5-week-old plants grown under CL intensity of ~75 µmol photons m^−2^ s^−1^ (12 h/12 h day/night cycle) were divided into two groups: a control group that stayed under CL and the second group that was exposed to FL in the same climate chamber (**Figure 1**). The light intensity in FL was changing between ~75 (CL background; ~280 s) and ~1,000 µmol photons m^−2^ s^−1^ (~20 s) during the light period. On the third day of the FL treatment, young and mature leaves (as defined by Schneider et al., 2019) were harvested from the plants 1 h (morning young, “MY” and morning mature, “MM”) and 11 h (end of day young, “EY”, and end of day mature, “EM”) after the light was turned on. Note that both morning and end-of-day samples were taken during the light phase and not in the dark phase of the 12 h/12 h light/dark regime. For each of the four combinations of leaf development stage × time of day (MY, MM, EY, EM), which are defined as the four different “samples” of the experiment, leaves were individually harvested from five biological replicate plants of CL and FL and immediately frozen in liquid nitrogen.

**Figure 2:**
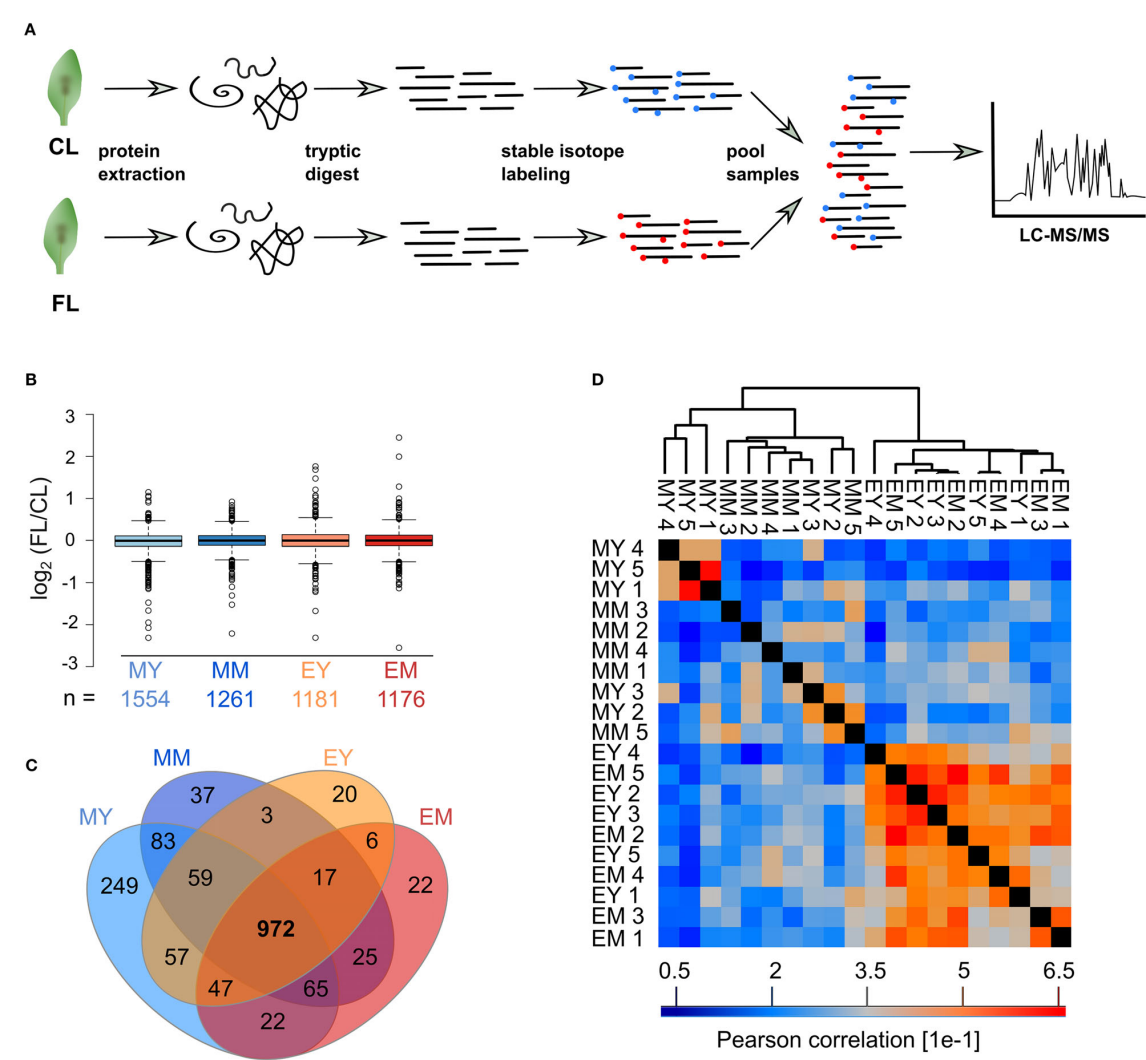
Proteomics workflow and overview of the FL experiment dataset. **(A)** Proteins were extracted, digested into peptides and CL and FL samples differentially stable isotope labeled with formaldehyde reagents before randomized pairwise pooling of FL and CL proteomes for analysis by nanoLC-MS/MS. **(B)** Median normalized FL/CL ratios of proteins quantified in atleast three of the five biological replicates of each sample. Whiskers extend to 5th and 95th percentile. **(C)** Overlap of proteins quantified in at least three out of five replicates in the four samples. **(D)** Pearson correlation of protein ratios between the biological replicates of the 972 proteins that were quantified in at least three replicates for every sample.

Proteins were extracted separately from each of the five biological replicates and digested with trypsin (**Figure 2A**). Subsequently, peptides of CL and FL were differentially labeled with stable isotope by reductive dimethylation before pairwise pooling of one FL and one CL probe of the same developmental stage and time point. Thus, there were five biological replicates of FL/CL comparison for each of the four samples (MY, MM, EY, and EM) for the proteome analysis. Spectrum to database matching identified a total of 2,313 proteins in the samples (**Supplementary Table 1**), of which 1,554, 1,261, 1,181, and 1,176 were reliably quantified in at least three out of the five biological FL/CL replicates of MY, MM, EY, and EM, respectively. At both leaf development stages and time points, FL-induced changes in protein abundance were very moderate, with more than 90% of the quantified proteins changing by less than 40% in abundance (**Figure 2B**). This is in line with the absence of visible stress symptoms and the relatively small changes in the transcript abundance previously found in young and mature leaves of *Arabidopsis* plants (Schneider et al., 2019) that were comparable with the leaves used in this study. In total, 972 proteins were reliably quantified in at least three replicates of all four samples (**Figure 2C**). Pearson correlation of FL/CL ratios of these 972 proteins revealed two distinct clusters among all individual replicates, namely, the morning and the end-of-day cluster, while young and mature leaves were not separated (**Figure 2D**). Notably, the correlation between the individual replicates of the end-of-day samples (EY and EM) was stronger than the correlation between those of the morning samples (MY and MM), indicating clearer and more consistent changes happening in leaf proteome at the end of day under the FL condition.

**Figure 3:**
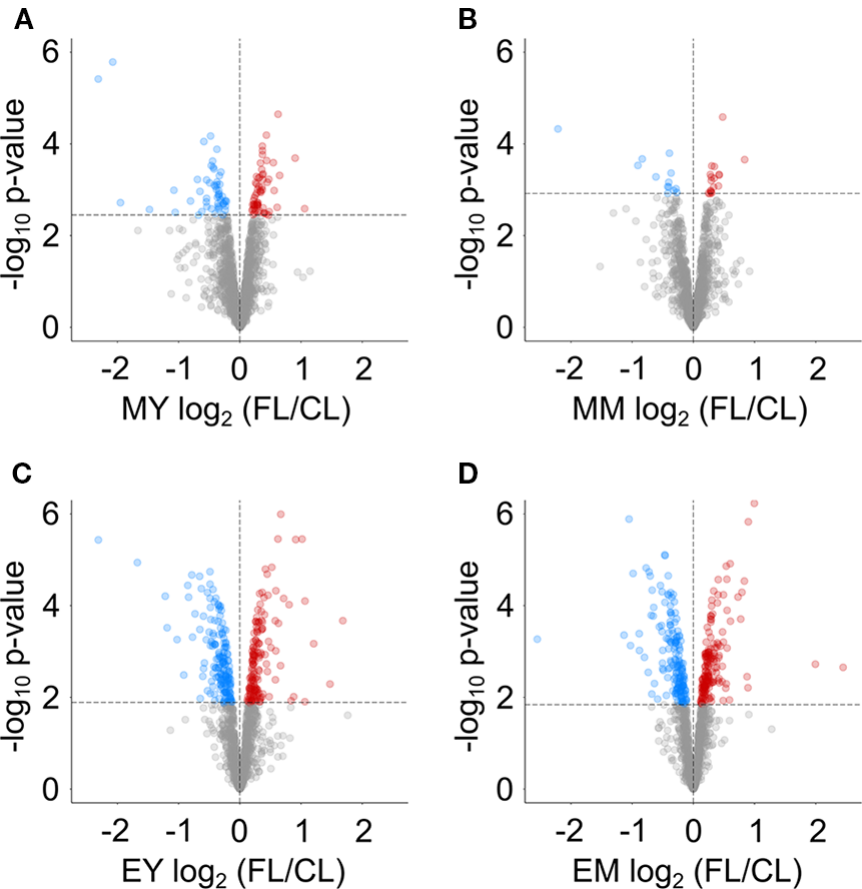
Volcano plots indicating proteins with significant changes in abundance in FL compared to CL at **(A)** MY, **(B)** MM, **(C)** EY and **(D)** EM. Dashed lines indicate the chosen significance thresholds with a Benjamini-Hochberg corrected FDR < 0.05. Blue circles indicate depleted, red circles accumulating proteins.

We next determined proteins with significantly altered abundance under FL using LIMMA-moderated t-test (Ritchie et al., 2015; Phipson et al., 2016) with a FDR-adjusted p-value of ≤0.05 as significance threshold (**Figure 3**). Even though the numbers of reliably quantified proteins and the FL/CL ratio distributions were comparable in all four samples (**Figure 2B**), only 112 and 31 proteins passed this threshold in MY and MM, respectively, whereas 341 and 321 proteins showed significant changes in EY and EM (**Figure 3**). These results provide a further support to greater proteome adjustments occurring in leaves after 11 h FL exposure (end of day) than after 1 h FL exposure (morning) on day 3.

### A Core Set of FL-Responsive Proteins in Leaves

**Figure 4:**
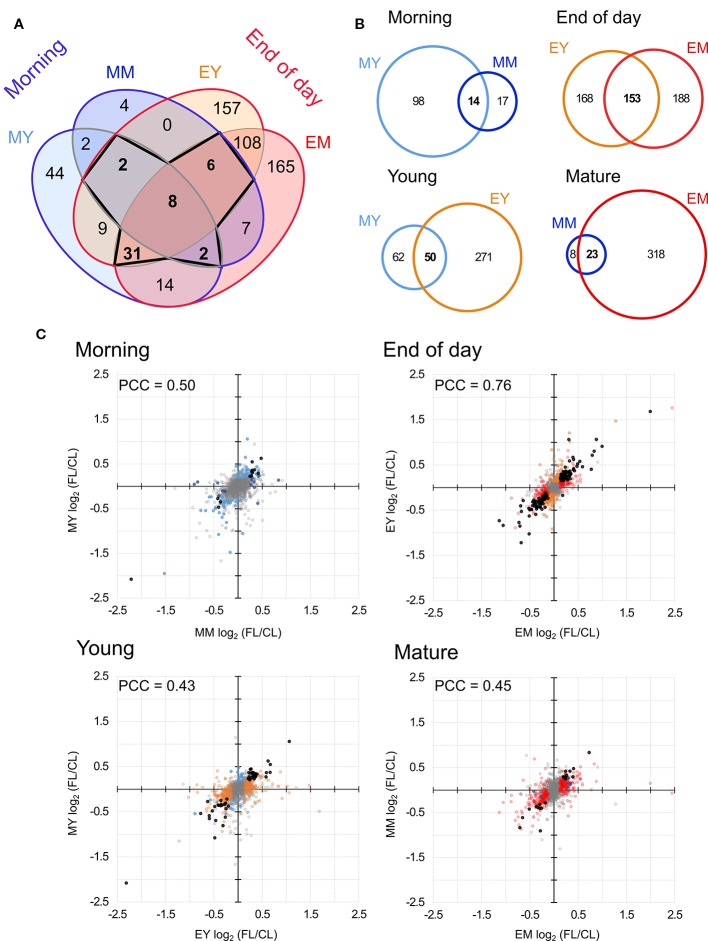
Comparison of proteins with significant FL-induced changes in abundance in each sample. **(A)** Overlap of significant changes in abundance in morning and at the end of day in both young and mature leaves. Bold numbers indicate the 49 proteins significantly changed in four or three samples. **(B)** Pairwise overlap of significant changes in protein abundance in the morning, at the end of day, in young leaves and in mature leaved. **(C)** Correlation of protein abundance ratio FL/CL between different samples. Colors indicate the sample in which the protein ratio was found to be significantly altered light blue, MY; dark blue, MM; orange, EY; red, EM. Black dots indicates significant change in both samples (overlap of Venn diagrams) and grey indicates no significant change. PCC, Pearson correlation coefficient.

Only eight proteins showed significant changes in abundance in all four samples under FL (**Figure 4A**, **Table 1**). Six of these underwent a significant increase under FL, namely, four chloroplast-located proteins fibrillin 1A (FBN1A), thiamine biosynthesis protein C (THIC), 31 kDa RNA-binding protein (RBP31), and a putative translation elongation factor (suppressor of variegation 3, SVR3), as well as cytosolic fructose-bisphosphate aldolase 5 (FBA5) involved in glycolysis and gluconeogenesis and mitochondrial glycine decarboxylase P protein 1 (GLDP1) catalyzing photorespiratory CO_2_ release. Two cytosolic glutathione S-transferases (GSTF10 and GSTU20) were the only proteins that declined in all samples under FL. Another 41 proteins showed significant increase or decrease in three out of four samples (**Figure 4A**). Of these, 31 and six did not pass the significance threshold in MM and MY, respectively, whereas only one did not pass in each of EY and EM. As was seen in **Figure 2D**, EY and EM had the largest overlap of the significantly changing proteins while much fewer proteins were common in young and mature leaves in the morning or in the same leaves between the two time points (**Figure 4A, B**). Still, a consistent trend of increase or decrease was observed for almost all 41 proteins in MY, MM, EY, and EM (**Table 2**), suggesting that these proteins responded to FL in a more or less similar manner, independently of time of day and leaf development stage. We therefore consider these 49 proteins in **Tables 1** and **2** as a core set of FL-responsive proteins in leaves during long-term acclimation to FL.

About a half of these proteins (26) were found to be increased and the other half (23) decreased after 3-d FL exposure. The analysis of associated GO terms indicated an enrichment of chloroplast proteins and enzymes involved in central carbon metabolism among the significantly increasing proteins, including those in carbon fixation, photorespiration, pentose phosphate pathway, glycolysis, and gluconeogenesis (**Supplementary Table 2**). In contrast, many proteins, which were less abundant in FL than in CL, were not localized in chloroplasts and no particular metabolic pathway was enriched (**Supplementary Table 3**).

Of the core set of 49 FL-responsive proteins, a putative transmembrane protein (AT3G07470) underwent the strongest increase of +85~110% in MY, EY, and EM (**Table 2**). In addition, young and mature leaves showed 45%~60% and 25%~40% increase, respectively, in the aforementioned cytosolic FBA5 and FBN1A in plastoglobuli at both time points (**Table 1**). One of the three isoforms of 3-deoxy-D-arabino-heptulosonate 7-phosphate synthase (DHS2), which catalyzes the first committed step of aromatic amino acid biosynthesis in the Shikimate pathway, also exhibited an increasing trend of +45%~50% (end of day) or +20%~30% (morning) (**Table 2**). On the other hand, a protein involved in guard cell outer cuticular ledge formation (FOCL1), which affects the size of guard cells and the aperture of stomatal pore (Hunt et al., 2017), was diminished by 75%~80% in both leaves at both time points, although the change was not statistically significant in EM. A marked decrease was also observed for a bacterial trigger factor-like protein (30%~40%) and cold- and dehydration-regulated protein 47 (COR47) (40%~45%) with an exception in MM and MY, respectively, in which these proteins showed a non-significant decrease or increase (**Table 2**). However, compared to the other three, MY had more pronounced decrease of plastid-encoded 10 kDa PSII reaction center protein psbH (−50%) and an adenylate kinase-like protein ADK1 (−40%) which monitors and interconverts ATP, ADP, and AMP in the cell. Interestingly, two catalase isoforms CAT2 and CAT3, which detoxify H_2_O_2_ in peroxisome, were both responding to FL but in the opposite directions, with CAT2 being 15%–25% more and CAT3 25%–35% less abundant under FL compared to CL.

### Time-of-Day-Specific Responses to FL

The Venn diagram (**Figure 4A**) pointed to a large number of additional proteins (430) which underwent significant changes in abundance only at the end of day, with 108 affected both in EY and EM. In contrast, only 50 proteins were significantly increased or decreased exclusively in the morning and no more than two proteins were shared by MY and MM. In order to quantify the similarities and differences among the four samples, we plotted changes in the protein abundance (FL/CL ratios) in the pairwise comparisons (**Figure 4B**). When data of all proteins quantified in at least three of the five biological replicates were included, the highest Pearson correlation coefficient (PCC) was found for the comparison of young and mature leaves at the end of day (**Figure 4C**). The correlation coefficient was much lower for the other three comparisons, consistent with the correlations found between the individual FL/CL replicates (**Figure 2D**). Together, the results of these analyses underline a higher similarity in FL-induced proteomic responses of young and mature leaves at the end of day than in the morning, and also distinct responses of the same leaves between morning and the end of day.

Proteins, which were significantly increased in both EY and EM but not in the morning, are enriched in chloroplast proteins and enzymes of central carbon metabolism (**Supplementary Table 4**), in much the same way as for the core set of significantly increasing proteins in FL (**Supplementary Table 2**). Thus, it seems that accumulation of certain chloroplastic and carbon metabolic proteins was constitutively enhanced in leaves under FL while other proteins increased more strongly towards the end of day. In addition, the proteins undergoing end-of-day-specific increase are also overrepresented by the components of oxidation-reduction, RNA binding and response to chemical or cold (**Supplementary Table 4**). Similarly, the GO terms, which are enriched in the proteins with end-of-day-specific decrease (**Supplementary Table 5**), are partly identical with those of the core set of significantly decreasing proteins (**Supplementary Table 3**), such as cytoplasm and cytosol or response to cadmium and inorganic substance. However, proteins with end-of-day-specific decrease were additionally associated with unique GO terms, e.g., sulfur-containing proteogenic amino acid metabolism, protein processing in endoplasmic reticulum, and ribosomes along with response to abiotic stimuli (temperature, heat) (**Supplementary Table 5**).

### Correlation Between FL-Induced Changes in Leaf Proteome and Transcriptome

Our recent RNA sequencing study identified 1,041, 266, 819, and 2,402 differentially expressed genes (> 30% changes in transcript abundance) in MY, MM, EY, and EM samples taken from the plants under the same FL and CL conditions (Schneider et al., 2019). Part of these gene expression responses were in line with the phenotypic changes observed at the level of growth and photosynthesis or pigments and other leaf metabolites. In order to make a comparison with the protein responses, we checked the FL-induced changes in protein abundance, focusing on the pathways reported in the transcriptome study (Schneider et al., 2019), namely, photosynthesis (light reactions, the Calvin-Benson cycle and their regulation), photorespiration and metabolism of pigments, prenylquinones and vitamin B6. **Supplementary Table 6** lists abundance ratios for selected proteins quantified in at least three replicates of all four samples, even if they did not meet the chosen significance thresholds. Generally, distribution of increasing or decreasing proteins is highly heterogenous among the pathways, suggesting both pathway- and component-specific regulation of protein abundance during long-term FL acclimation.

For example, the components of PSII and PSI, especially some subunits of oxygen evolving complex in the morning and PSI reaction center in mature leaves, tended to decrease under FL (**Supplementary Table 6**). In contrast, the components of intersystem and cyclic electron transport were mostly increased, as were those of thylakoid ATP synthase. Many thioredoxins (TRXs) and redox regulatory enzymes of chloroplasts were also more likely to increase than decrease in FL. The Calvin-Benson cycle and photorespiratory enzymes seemed to undergo small but concerted increase, which more frequently passed our significance criteria in young leaves than in mature leaves. The exceptions to this increase were the small subunit of Rubisco (RBCS) and catalase 3 (CAT3), which showed minor or strong decrease, respectively. Apart from the suppression of protochlorophyllide reductase B (PORB) accumulation, the abundance of chlorophyll biosynthetic enzymes changed rather weakly while several enzymes in the isoprenoid and tocopherol biosynthetic pathways increased. Similarly, the protein levels were also increased for two isoforms of pyridoxal 5’-phosphate synthase subunit 1 (PDX1).

**Figure 5:**
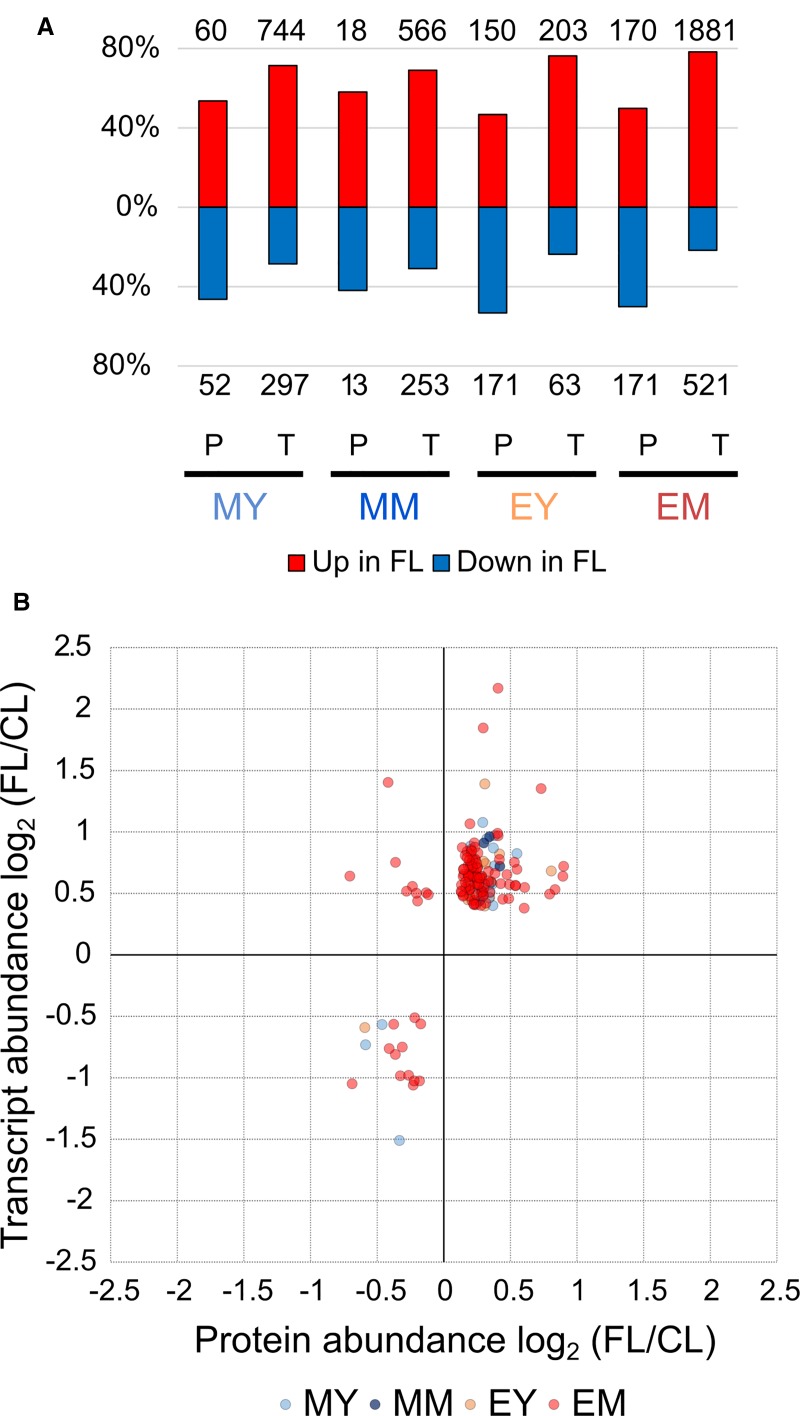
Comparison of the FL response on the protein and transcript levels. **(A)** Percentage of significant increase or decrease in protein and transcript abundance in FL. Numbers above and below the bars show absolute numbers of proteins/transcripts found in each sample. **(B)** Quantitative correlation between significant changes in protein and transcript abundance. Twenty proteins/genes were significantly changed in MY, 5 in MM, 14 in EY, and 123 in EM.

The changes in protein abundance observed in these pathways correspond largely but not entirely to the gene expression changes described in our previous study (Schneider et al., 2019). In fact, we found a universal shift in the relative proportions of significantly increasing and decreasing proteins and genes (**Figure 5A**). Proteins exhibiting significantly increased and reduced abundance under FL were in nearly equal proportions (approximately 50%) in each of the four samples, whereas upregulation was clearly the predominant response at the level of gene expression. Since the numbers of significantly increasing or decreasing proteins and genes detected by the proteome and transcriptome analyses differed substantially, the intersections between the global changes of proteome and transcriptome are not large (**Supplementary Figure 2**). In total, 145 unique proteins/genes were significantly and concurrently changing in abundance in the corresponding leaves at the same time points.

Counting also the responses of the same proteins/genes in multiple samples, we obtained 169 individual relations of protein and transcript abundance for pairwise comparison, mostly from EM (**Figure 5B**). The majority of the proteins/genes (138) clustered in the upper right corner of the plot, representing parallel increase in both protein and transcript under FL (**Supplementary Table 7**). This included many components associated with GO terms related to chloroplast transcription and translation, photosynthesis and photorespiration, plastid organization, and oxidation-reduction process (**Supplementary Table 8**). For most of them, the measured increase was greater at the mRNA level than at the protein level. Only seven had larger FL/CL ratios for protein abundance, of which three are in chloroplasts: ferredoxin:thioredoxin reductase catalytic subunit (FTRB), atypical Cys/His-rich thioredoxin 2 (ACHAT2), and redox-regulated glucose-6-phosphate dehydrogenase 1 (G6PD1). The other four are peroxisomal acyl-activating enzyme 7 (AAE7) having short-chain acetyl-CoA synthetase activity, cytosolic carotenoid cleavage dioxygenase 1 (CCD1), an ABC transporter family protein (ABCB28) and an unknown transmembrane protein (AT2G05310; AT4G13500). The accumulation of these seven proteins was enhanced in EM while AAE7 was also increased in EY (**Supplementary Table 7**).

In contrast, only 16 proteins/transcripts clustered in the lower left corner (**Figure 5B**) indicating concomitant downregulation in FL (**Supplementary Table 9**). This included proteins linked to peroxisomal and cytosolic ROS processing (CAT3; dehydroascorbate reductase 1, DHAR1; annexin D1), protein folding in endoplasmic reticulum (protein disulfide isomerase-like proteins PDIL1-1 and PDIL2-1; HSP70-family proteins BIP1 and HSP70-17; calreticulin 1, CRT1), and chlorophyll biosynthesis (PORB). In most cases, the decrease was larger at the mRNA level (**Supplementary Table 9**).

Intriguingly, nine data points (all from EM) appeared in the upper left corner, showing a decrease in protein abundance despite increased transcript abundance (**Figure 5B**). Eight of these proteins are (or are predicted to be) localized in chloroplasts (**Table 3**): Rubisco small subunit 1B (RBCS1B) and glyceraldehyde-3 phosphate dehydrogenase A1 (GAPA1) in the Calvin-Benson cycle, MAR-binding filament-like protein1 (MFP1) associated with nucleoids and thylakoids, 50S ribosomal protein L13 family protein (EMB1473), threonyl-tRNA synthetase (EMB2761), plasma membrane fusion protein (AT5G42765), bacterial trigger factor (AT2G30695), and saccharopine dehydrogenase (AT1G50450). The only non-chloroplastic protein in this group, sinapoylglucose:malate O-sinapoyltransferase (SNG1 or SCPL8), is an enzyme which synthesizes a UV screening compound sinapoyl malate in leaves. Diagonal to these, no single protein/gene was found in the lower right corner combining protein increase with transcript decrease (**Figure 5B**). Together, the results in **Figure 5B** suggest that FL-induced changes in transcript abundance are mostly reflected in the changes in protein levels, although some proteins may accumulate to lower levels despite increased transcription.

## Discussion

Concordant with the recent transcriptome study (Schneider et al., 2019), our quantitative proteome analysis revealed distinct acclimatory changes in protein composition and abundance of MY, MM, EY, and EM after 3-d in the FL condition (**Figure 4**), underpinning developmentally and temporally heterogeneous adjustments of leaf proteome within a small rosette of Arabidopsis during acclimation. At the protein level, the FL responses of young and mature leaves were greater and also more similar at the end of day than in the morning (**Figures 3** and **4B**), whereas their gene expression responses were overlapping more strongly in the morning (Schneider et al., 2019). While we found partly comparable changes in transcript and protein abundance, there were also differences in their responses, such as the relative importance of downregulation being greater at the protein level than the transcript level (**Figure 5A**) and the decline in protein abundance despite upregulation of gene expression seen for some proteins (**Figure 5B**). Obviously, FL acclimation entails post-transcriptional control involving regulation of translation and protein turnover as proposed for HL acclimation (Oelze et al., 2014; Li et al., 2018). Below, we discuss key findings of our study and their implications for mechanisms of long-term FL acclimation.

### Acclimation to FL Entails Both Constitutive and Dynamic Adjustments of Proteome

In agreement with the transcript data (Schneider et al., 2019), we found both consistent and distinct proteome alterations in the four samples during acclimation to FL (**Figure 4**). Similar to the transcriptional changes and consistent with the lack of visible stress symptoms (Schneider et al., 2019), changes in protein abundance were very moderate, with few proteins exhibiting more than 50% changes in any sample. The core set of 49 proteins (**Tables 1** and **2**) showing significant changes in abundance in three or four of the four samples (MY, MM, EY, and EM) suggest constitutive responses to FL shared by young and mature leaves in the morning and at the end of day. These constitutive responses included enhanced accumulation of proteins involved in formation of plastoglobuli and stress response (FBN1A, FBN1B), CEF around PSI (NDHM), photorespiration (GLDP1, SGAT, CAT2, GDCST), and glycolytic enzymes (FBA5, CFBP). This pattern is consistent with the previously observed alterations in leaf transcriptome and phenotype which were associated with reduced linear electron transport, increased photoprotection, decreased sugar and starch accumulation, and growth reduction (Alter et al., 2012; Schneider et al., 2019). In contrast, several glutathione S-transferases (GSTU20, GSTF9, GSTF10), cold-regulated proteins (COR15B, COR47) and proteins involved in translation (bacterial trigger factor, elongation factor EF1B, ribosomal protein RPL16) were consistently depleted under the FL condition (**Tables 1** and **2**).

In addition to these constitutive changes, only a relatively small number of proteins exhibited significant changes in abundance under FL in the morning (**Figure 4**). In contrast, many proteins (430) showed significant changes in abundance in young and/or mature leaves at the end of day under FL (**Figures 3** and **4**), including proteins of the same functional categories as the core set, but also those associated with other stress-related GO categories such as “protein processing in endoplasmic reticulum” and “response to abiotic stimuli (temperature, heat)”. Although similar patterns were also recognized in the corresponding leaves in the morning, the changes became more significant at the end of day, suggesting additional stimuli or signals generated in the course of the day under FL to strengthen these responses.

Especially striking is the significant accumulation of photorespiratory enzymes in EY and decrease in PSI complex subunits in EM (**Supplementary Table 6**). Upon sudden increase in light intensity, the components of light reactions respond more rapidly than the Calvin-Benson cycle, which in turn is quicker than stomatal guard cell movement. Consequently, CO_2_ concentration transiently drops inside the leaf (Vialet-Chabrand et al., 2017) and hence also in chloroplast stroma during light intensity fluctuation. This was shown to boost photorespiration even in leaves of tobacco plants grown under full sunlight (Huang et al., 2015). The concerted upregulation of many photorespiratory proteins/genes found in this study and our previous RNA sequencing analysis under the FL condition (Schneider et al., 2019) is in line with limitation of photosynthetic carbon fixation by temporally low CO_2_ availability in C3 plants. Indeed, a recent study in cassava reported a major impact of stomatal conductance to limit dynamic photosynthesis in FL (De Souza et al., 2020). Signals that trigger the upregulation of photorespiratory genes and proteins under FL are not yet known.

It has been suggested that PSI is susceptible to photoinhibition and oxidative damage when excess electrons arrive from PSII (Tikkanen et al., 2012). Thus, PSI needs to be protected by downregulating PSII through NPQ and alleviating acceptor side limitation by, for example, CEF and photorespiration under FL conditions. In accordance, the Arabidopsis leaf transcriptomes showed concomitant upregulation of many genes related to these processes (Schneider et al., 2019) and our proteome analysis confirmed an increase in protein abundance for some of them (**Table 2** and **Supplementary Table 6**). In particular, mature leaves exhibited concurrent upregulation of NDH-like complex genes at the end of day, suggesting a role of NDH-dependent CEF in these leaves under our FL condition (Schneider et al., 2019). The accumulation of the NDH-like complex and its chloroplast-encoded subunits (ndhA and ndhF) has been shown to increase in barley leaves under photooxidative conditions or following H_2_O_2_ treatment (Martín et al., 1996; Casano et al., 2001).

Leaves exposed to FL had lower protein levels of PS I subunits (**Supplementary Table 6**), which supports the notion of PSI photoinhibition and degradation. Far-red (FR) light protects PSI against photoinhibition under FL (Kono et al., 2017). The protective effects of FR illumination were substantially reduced in mutants lacking NDH-dependent CEF, suggesting the contribution of this alternative electron transport pathway in natural FR-rich FL environments (Kono et al., 2017). Our growth light conditions had very high red-to-FR ratio (4 to 5; **Supplementary Figure 1**) compared to natural sunlight (~1.2) or canopy shade (0.1 to 0.2) (Franklin, 2008), which could have exacerbated PSI photoinhibition in our experiments.

### Acclimatory Adjustment of Protein Abundance Involves Pathway- and Protein-Specific Changes in Synthesis and Degradation

After 3-d exposure to FL, most changes in protein abundance were rather small and challenging to detect even with the precision of stable isotope-based quantification such as the chemical labeling method employed here (Altelaar et al., 2013). To identify broader trends, we therefore inspected changes in protein abundance in selected protein complexes and pathways of interest, irrespective of whether they passed the chosen significance criteria or not (**Supplementary Table 6**).

Among the protein complexes involved in photosynthetic electron transport, the abundance of PSI subunits tended to decrease in FL as discussed above, while cytochrome b_6_f and the NDH-like complexes appeared to accumulate (**Supplementary Table 6**), which is in accordance with the necessity to control electron flows during light fluctuation. In comparison, PSII abundance changed little as the photoprotective NPQ capacity increased to downregulate PSII activity in both young and mature leaves (Alter et al., 2012; Schneider et al., 2019). However, individual protein subunits of these large complexes showed highly specific changes in FL. For instance, psbH, a small one-helix phosphoprotein subunit of PSII reaction center which is required for accumulation of CP47 (Levey et al., 2014), showed a significant reduction (50%–84% of the levels in CL) in protein abundance in nearly all samples (**Table 2**). Likewise, NDHM, a unique photosynthetic subunit of the NDH-like complex which may stabilize the conserved core component NDHK (Yamamoto et al., 2016), was consistently and significantly increased in FL (+25%–35%), whereas the FL responses of other subunits were smaller and/or not significant except PNSB1 which increased by ~10% from morning to the end of day in both young and mature leaves (**Supplementary Table 6**). These results suggest that acclimation may specifically modulate the abundance of subunits and enzymes which determine the assembly, stability, or activity of protein complexes (e.g., psbH, NDHM).

Protein synthesis and degradation constitutes a major resource allocation for the plant (Nelson and Millar, 2015; Li et al., 2017). This is reflected in markedly reduced rates of protein translation in the dark (Piques et al., 2009; Pal et al., 2013) and dependency of translation on carbon availability (Ishihara et al., 2015). Thus, transcriptional changes observed early in the morning (Schneider et al., 2019) may not yet be translated into changes in protein abundance in the same samples. This could provide an additional explanation for the much fewer changes in proteome observed in MY and MM compared to EY and EM (**Figures 3** and **4**).

Remarkably, FL predominantly upregulated transcription of various genes in all MY, MM, EY, and EM, with relatively few genes repressed in expression (Schneider et al., 2019). In contrast, the proportion of proteins with increased and reduced abundance was approximately equal (**Figure 5A**), indicating greater importance of post-transcriptional mechanisms for decreasing the abundance of individual proteins during acclimation. Furthermore, even for genes/proteins found upregulated both on transcript and protein levels, the relationship between the changes in abundance of transcript and protein was specific for each gene/protein pair (**Figure 5B**), suggesting protein-specific fine-tuning. Most severely affected were nine mainly plastid-localized proteins, showing reduced abundance despite increased transcript levels in EM (**Table 3**). These included proteins involved in plastid translation (a putative bacterial trigger factor, threonyl-tRNA synthetase, and a 50S ribosomal protein) and CO_2_ assimilation (RBCS1B, GAPA1). It is conceivable that the mild but chronic photooxidative conditions in our FL treatment trigger these changes in chloroplast proteins at the end of day, presumably by enhancing protein degradation and/or inhibiting translation.

To clarify the relative contributions of protein synthesis and degradation to the observed changes in the abundance of different proteins, future studies should aim to determine the rates of protein translation, e.g., by ribosome profiling (Juntawong et al., 2014; Mazzoni-Putman and Stepanova, 2018), and degradation, e.g., using metabolic stable isotope labeling (Li et al., 2017), during acclimation to FL conditions.

### Towards a Systems-Level Understanding of Proteome Adjustment in FL Acclimation

Previous studies have characterized changes in plant phenotype under FL conditions, including growth, photosynthetic performance, gene transcription, metabolome, and the abundance of selected proteins (Yin and Johnson, 2000; Alter et al., 2012; Huang et al., 2015; Kaiser et al., 2016; Vialet-Chabrand et al., 2017; Schneider et al., 2019). Our study now provides a first overview of dynamic acclimatory changes in the proteomes of Arabidopsis leaves during long-term acclimation to FL.

A still unknown factor in FL conditions is the extent of oxidative damage and protein modifications caused by excess light, excess electrons, and ROS. If FL intensifies these inhibitory effects, costs of damage and repair would increase in parallel with acute demand for protection and alternative pathways to mitigate the stress. The changes in leaf proteome and transcriptome observed in our FL conditions support this scenario. Concomitant upregulation of these and related processes will necessitate energy and resource, which, together with increased loss of light energy and fixed carbon by NPQ and photorespiration, would restrict the resource availability for growth. In fact, cell cycle- and growth-related genes were downregulated in young and mature leaves accumulating less starch, less sugars, and less amino acids under FL (Schneider et al., 2019).

To fully unravel the mechanisms of acclimatory proteome adjustments, it is necessary to overcome technical limitations. Here, we identified 2,313 proteins, but only 972 (or 42%) of these were reliably quantified in all four samples. Due to the stochastic nature of mass spectrometry-based experiments, the absence of quantitative information does not mean the absence of the protein, which impaired comparison across samples. In future studies, this may be overcome by targeted and/or data-independent mass spectrometry approaches that increase proteome coverage and precision of quantification (Navarro et al., 2016; Ludwig et al., 2018). Separate preparation of soluble and membrane protein fractions may further improve proteome coverage particularly of the latter fraction and reveal changes in physiologically important protein complexes and transporters in thylakoid membranes and chloroplast envelopes (Ferro et al., 2010). Furthermore, a variety of oxidative protein modifications, which may be triggered by photooxidative stress under FL to alter protein stability (Galetskiy et al., 2011) and enzymatic activities (Stroher and Dietz, 2008; Zaffagnini et al., 2012) or to mediate signaling (Noctor et al., 2018), may be identified by selective redox proteomics (Astier et al., 2011; Mock and Dietz, 2016). It is well known that such post-translational modifications can alter enzyme activity without changes in protein abundance.

## Concluding Remarks

Long-term acclimation to FL involves both constitutive and dynamic adjustments of the proteome in young and mature leaves of Arabidopsis. Dynamic adjustments, mostly occurring during the daytime, may allow flexible and iterative responses to highly variable and unpredictable changes in natural light environments. Constitutive adjustments, on the other hand, are more rewarding when the condition is not changing substantially from day to day. While some major components of FL acclimation, such as upregulation of CEF and photorespiratory pathway, engage changes in both transcript and protein abundance, post-transcriptional regulation seems to modulate the relationship between these changes in a protein-specific manner. The mechanisms of proteome acclimation in FL await further investigations, including the analyses of translation and proteolysis or post-translational protein modifications.

## Data Availability Statement

The datasets generated for this study can be found in the PRIDE partner repository of the ProteomeXchange Consortium (https://www.ebi.ac.uk/pride/archive/) with the following accession number: PXD015330.

## Author Contributions

SN and M-OB performed shotgun proteomics experiments. TS performed the plant stress experiment and harvested material. SN, SM, and PH analyzed and interpreted the data and wrote the manuscript. SM and PH designed and supervised the project.

## Funding

This work was in part supported by funding from the European Research Council under the European Union’s Horizon 2020 research and innovation program (starting grant 639905 “ProPlantStress” to PH) and by the Deutsche Forschungsgemeinschaft (IRTG1525 to SM).

## Conflict of Interest

The authors declare that the research was conducted in the absence of any commercial or financial relationships that could be construed as a potential conflict of interest.
