# Supplementary material for: Photoprotective Acclimation of the Arabidopsis thaliana Leaf Proteome to Fluctuating Light
Source: Front Genet. 2020 Mar 5;11:154. doi: 10.3389/fgene.2020.00154 (PMC7066320; doi:10.3389/fgene.2020.00154)
Supplement: Supplementary file 2 [file Presentation_1.pdf]

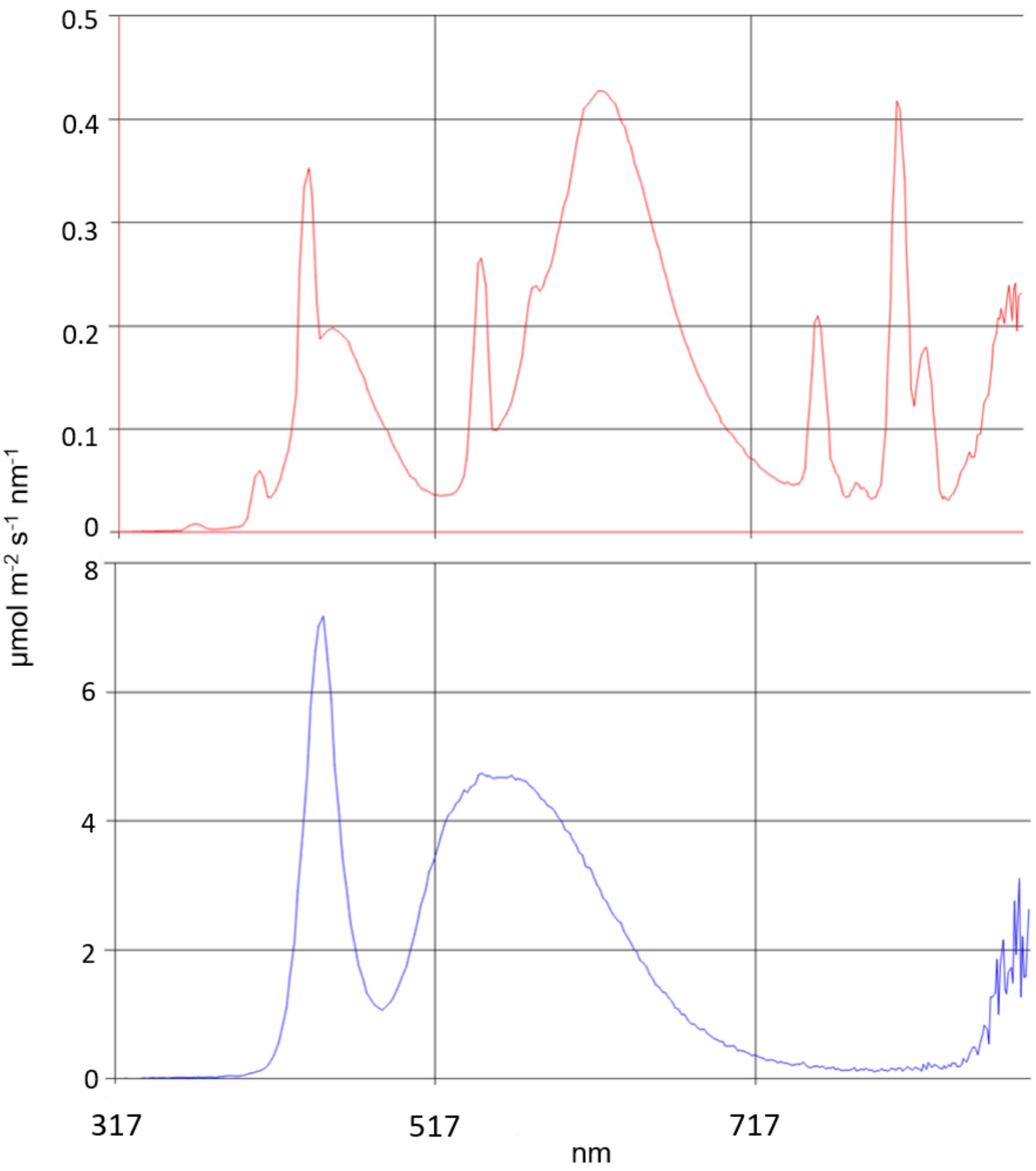

Supplementary Figure 1: Spectra of the fluorescent tubes used for growth and constant light conditions (CL, top panel) and of the white LEDs used for high-intensity fluctuating light pulses (FL, bottom panel).

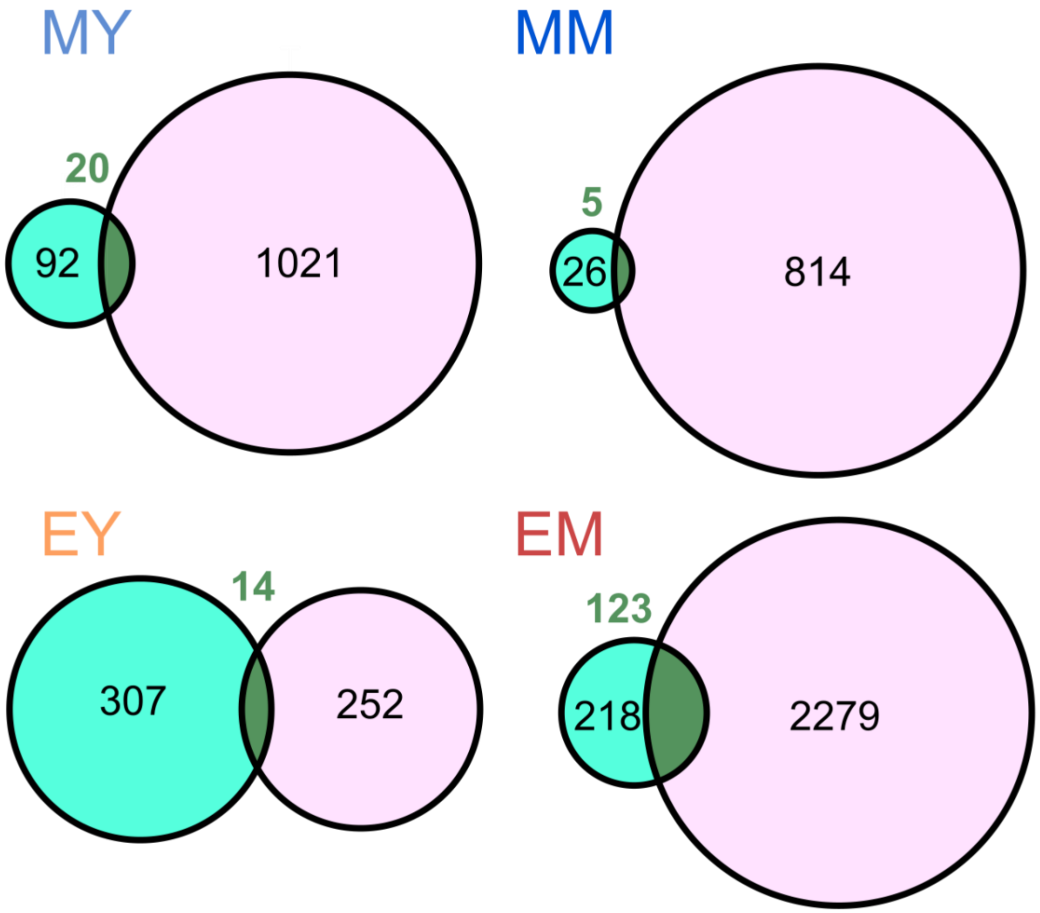

Supplementary Figure 2: Overlap of proteins and transcripts with significant changes in abundance during acclimation to FL. Teal indicates proteins, pink transcripts and bold green genes with significant changes on both the proteins and transcripts level. MY, young leaves in the morning (1h after lights on); MM, mature leaves in the morning; EY, young leaves at the end-of-day (after 10h exposure to fluctuating light, 1 h before lights out,); EM, mature leaves at the end-of-day.
